# Comparison of whole-body MRI, bone scan, and radiographic skeletal survey for lesion detection and risk stratification of Langerhans Cell Histiocytosis

**DOI:** 10.1038/s41598-018-36501-1

**Published:** 2019-01-22

**Authors:** Jeong Rye Kim, Hee Mang Yoon, Ah Young Jung, Young Ah Cho, Jong Jin Seo, Jin Seong Lee

**Affiliations:** 1 Department of Radiology, Dankook University Hospital, Cheonan-si, Chungcheongnam-do South Korea; 2 Department of Radiology and Research Institute of Radiology, Asan Medical Center, University of Ulsan College of Medicine, Seoul, South Korea; 3 Department of Pediatrics, Asan Medical Center Children’s Hospital, University of Ulsan College of Medicine, Seoul, South Korea

## Abstract

Accurate risk stratification according to the extent of Langerhans cell histiocytosis (LCH) determined on whole-body evaluation is important for determining the treatment plans and prognosis in patients with LCH. This study aimed to compare the lesion detectability and the accuracy of risk stratification of skeletal survey, bone scan, and whole-body magnetic resonance imaging (WB-MRI) in patients with LCH. Patients with newly-diagnosed LCH who underwent all three imaging modalities were retrospectively included (n = 46). The sensitivity and mean number of false-positives per patient for LCH lesions, and the accuracy of risk stratification of each modality were assessed. WB-MRI had significantly higher sensitivity (99.0%; 95% confidence interval, 93.2–99.9%) than skeletal survey (56.6%; p < 0.0001) and bone scan (38.4%; p < 0.0001) for LCH lesions, and there were no significant differences in the number of false-positives per patient (p > 0.017). WB-MRI tended to have higher accuracy for the risk stratification than skeletal survey and bone scan (concordance rate of 0.98, 0.91, and 0.83, respectively), although the differences were not significant (overall p-value 0.066). In conclusion, WB-MRI had higher detectability for LCH lesions than skeletal survey and bone scan, while the three whole-body imaging modalities had comparable accuracy in the initial risk stratification of LCH.

## Introduction

The treatment options and prognosis for Langerhans cell histiocytosis (LCH) vary depending on the extent of the disease. Radiographic skeletal survey and bone scan have been used for the evaluation of the extent of LCH^1,2^; however, these have limitations in regard to the evaluation of extra-skeletal involvement of LCH. Positron emission tomography (PET) can detect LCH lesions in bones and soft tissue with greater accuracy than can a skeletal survey and bone scan^3–5^; however, PET has the disadvantage of radiation exposure.

Whole-body magnetic resonance imaging (WB-MRI) has been used for initial evaluation of disease extent and follow-up in various oncologic indications. WB-MRI has good soft tissue contrast, which is helpful in evaluating the extent of skeletal and extra-skeletal lesions, without subjecting the patient to ionizing radiation^6,7^. However, there are only two previous studies that report on the usefulness of WB-MRI in patients with LCH^8,9^, and these both had a small number of patients.

The purpose of our study was to compare the lesion detectability of skeletal survey, bone scan, and WB-MRI, and to determine the accuracy of risk stratification in patients with newly-diagnosed LCH.

## Materials and Methods

The institutional review board of Asan Medical Center approved this retrospective study and waived the requirement for informed consent. All experiments were performed in accordance with relevant guidelines and regulations. The methodology and reporting of this study followed the STAndards for Reporting Diagnostic accuracy studies (STARD) guideline^10^.

### Patients

**Figure 1:**
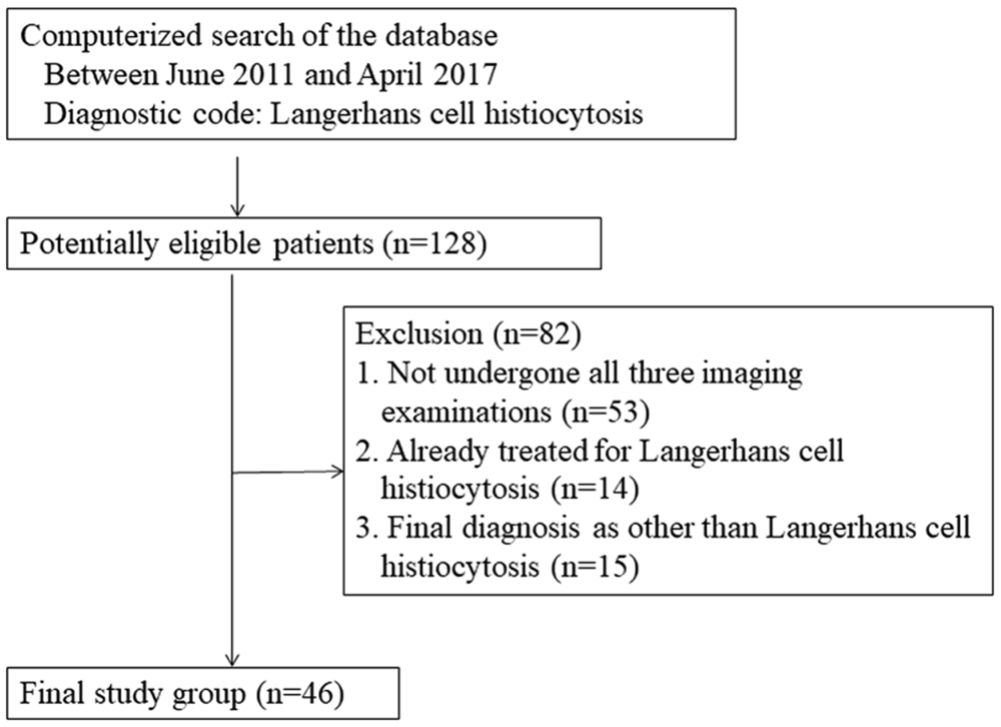
Flow chart of the patient selection process.

Patients with pathologically-confirmed LCH and who were referred to our tertiary referring hospital to undergo initial risk stratification and management were included in this study. A systematic computerized search of the hospital database was performed to identify eligible patients under the diagnostic code of LCH who presented between June 2011 and April 2017. The inclusion criteria were as follows: (1) patients with pathologically-confirmed LCH; (2) patients who were not previously treated for LCH; (3) patients who underwent all skeletal survey, bone scan, and WB-MRI, all of which were performed prior to initiation of treatment; and (4) patients who underwent imaging follow-up during the study period and had available imaging data. A total of 128 potentially eligible patients were identified, with 82 patients being subsequently excluded, as 53 of them had not undergone all three imaging examinations, 14 were referred to our hospital after receiving treatment, and 15 did not have LCH as the final diagnosis (Fig. 1).

### Image Acquisition

The radiographic skeletal survey, a series of radiographs covering the entire skeleton^11,12^, was performed using a Definium 8000 system (GE Healthcare, Chalfont St. Giles, UK). The skeletal survey imaging protocol covered the whole body as follows: chest (posteroanterior and lateral views), ribs (anteroposterior [AP] and both oblique views), skull (AP, both lateral, and Townes views), whole spine (AP and lateral views), and bilateral whole upper and lower extremities (AP and lateral views). The kVp ranged between 60 and 80, according to the patient’s age, body size, and the area imaged. The mA range was adjusted to between 40 and 100, according to the kVp.

Bone scans were performed using a large field-of-view gamma camera (Symbia E, Siemens) after an intravenous bolus injection of ^99m^Tc- 3,3-diphosphono-1,2-propanedicarboxylic acid.

WB-MRI was performed using a 3 T (Ingenia, Philips Medical Systems, Best, The Netherlands) or 1.5 T MR system (Achieva, Philips Medical Systems) with a dedicated multi-channel multi-element surface coil. Images were obtained using three to six subsequent table positions to cover the head to the toes, depending on the body dimensions. All WB-MRI examinations included coronal and sagittal short tau inversion recovery (STIR) images and coronal non-enhanced T1-weighted fast spin echo images. Post-contrast scans were performed with coronal three-dimensional fat-suppressed T1-weighted gradient echo imaging after intravenous administration of gadoterate meglumine (Dotarem®, Guerbet, Roissy CdG, France). Since March 2016, fat-suppression for the post-contrast scans was performed using a modified Dixon technique. All scans were taken during the free breathing state. Detailed parameters of the pulse sequences are presented in Table S1 of the Supplementary Materials. We evaluated the time interval between the three whole-body imaging modalities.

### Evaluation of lesion detectability

The skeletal survey, bone scan, and WB-MRI findings were retrospectively reviewed in consensus by two radiologists (H.M.Y. and J.R.K. with 8 and 6 years of clinical experience in radiology, respectively). Any discrepancy between the two radiologists was resolved by a third radiologist (J.S.L. with 28 years of clinical experience in radiology). The skeletal survey, bone scan, and WB-MRI of any given subject were reviewed separately, with the reviews being performed in random order, and with the reviewers blinded to the results of the other whole-body imaging modalities and the patient’s clinical data, except for the diagnosis of LCH.

Images were evaluated for the presence and number of skeletal and extra-skeletal lesions. Each detected lesion was categorized according to its anatomic location. The criteria used for evaluating the involvement of LCH on imaging findings based on previous studies is described in Supplementary Materials^13–21^. LCH lesions that had already been removed by excisional biopsy for diagnosis, and those in the skin, were excluded from the evaluation of the detectability of LCH lesions, even though postoperative changes may have been visible on the whole-body imaging modalities.

### Risk Stratification of Langerhans Cell Histiocytosis

All included patients were classified into one of four risk groups based on a modification of the current classification of LCH by the Histiocyte Society as follows: (a) Single system LCH (SS-LCH) without multiple bone lesions (MBL) or CNS risk lesions (CRL); (b) SS-LCH with MBL or CRL; (c) Multisystem LCH (MS-LCH) without risk organ involvement (RO); and (d) MS-LCH with RO. CRL included vertebral lesions with a intraspinal soft tissue component, and lesions in temporal, mastoid, sphenoid, zygomatic, ethmoid, maxilla bones, the orbit, paranasal sinuses, or cranial fossa. RO included liver, spleen, and the hematopoietic system^22–26^. Two radiologists (H.M.Y. and J.R.K.) performed risk stratifications for three separate sessions according to the findings of each whole-body imaging modality (skeletal survey, bone scan, and WB-MRI) in each patient. The reviewers were blinded to the imaging findings of the other whole-body imaging modalities, but not to the biopsy site where LCH was proved, or the imaging findings of a specific region that were obtained for evaluation of the initially presented symptoms before the diagnosis of LCH. For example, abnormal pituitary stalk thickening was found on sellar MRI obtained before pre-treatment whole-body work-up, this pituitary lesion was counted as an involved site in the risk stratification, even though this lesion was not visualized on the skeletal survey or bone scan. In addition, in cases where an LCH lesion had already been removed by excisional biopsy for diagnosis and/or treatment, the lesion was included as LCH involvement in each session of risk stratification, even though such lesions were no longer seen on the whole-body imaging modalities.

### Reference Standards

Initial diagnosis of LCH was histopathologically confirmed by core needle biopsy or open excisional biopsy of a particular lesion. Although biopsy was required to confirm the diagnosis, it may not have been possible to perform a biopsy on all of the suspected LCH lesions within a single patient. Consequently, the diagnosis of each suspected LCH lesion was made on the basis of clinical and imaging follow-up, with the consensus of two radiologists (A.Y.J. and Y.A.C. with 14 and 22 years of clinical experience in radiology respectively). All available imaging studies (region specific MRI or computed tomography [CT], ultrasound, PET-CT, skeletal survey, bone scan, and WB-MRI) were used for determining the final risk group as a reference standard.

### Statistical analysis

Quantitative variables are presented using descriptive statistics such as the mean ± standard deviation (SD) or the median and range.

The detectability of LCH lesions on each whole-body imaging modality was evaluated as the sensitivity and mean number of false-positives per patient, as all study subjects were patients with LCH, and thus there were no true-negative lesions. The detectability of skeletal lesions only was also evaluated. Logistic regression using a generalized estimating equation (GEE) was performed to compare the sensitivities of the whole-body imaging modalities, with this technique being able to account for the clustered data containing multiple observations per patient. Poisson regression analysis was performed to compare the mean number of false-positives per patient between the whole-body imaging modalities.

The accuracy of the initial risk stratification using each whole-body imaging modality was assessed using the concordance rates between the final risk groups and the risk groups classified by each of the whole-body imaging modalities, as well by using weighted kappa with the following scale: a weighted kappa value of 0 to 0.2, slight agreement; 0.21 to 0.4, fair agreement; 0.41 to 0.6, moderate agreement; 0.61 to 0.8, substantial agreement; and 0.81 to 1, almost perfect agreement^27,28^. GEE was also used to compare the concordance rates of risk stratification using the different whole-body imaging modalities. All analyses were performed using SAS v 9.3 (SAS Institute, Cary, NC, USA).

## Results

### Baseline Characteristics of the Patients and Disease Distribution

A total of 46 patients (21 male and 25 female patients, mean age ± SD, 10.6 ± 13.3 years; range, 0.1–55 years) were included in the study. The disease extent and the distribution of disease across the patients are presented in Tables S2 and S3 in the Supplementary Materials. The majority of patients had single system LCH (n = 36/46, 78.3%), with the skeletal system being the most commonly involved single system (n = 32/36, 88.8%). In patients with multisystem LCH (n = 10/46, 21.7%), four patients had only extra-skeletal lesions and seven patients had both skeletal and extra-skeletal lesions. There were nine patients less than 1 year of age, and more than half of them had multisystem LCH (n = 5/9, 55.6%).

### Lesion Detectability on skeletal survey, bone scan, and WB-MRI

A total of 105 lesions were found in 38 patients on the three whole-body imaging modalities. Of these, 99 lesions were considered as true LCH lesions, but six lesions turned out to be pseudolesions on clinical and imaging follow-up (Table 1). The mean ± SD of the time interval between the first and last whole-body imaging modality in each patient was 3.20 ± 3.35 days (range: 1–15 days).

**Table 1:** Distributions of all lesions detected on the whole-body imaging modalities (n = 105, including six pseudolesions).

True-positive lesions (n = 99)		False-positive lesions (n = 6)
Skeletal lesions (n = 81/99, 81.82%)	Extra-skeletal lesions (n = 18/99, 18.18%)	Skeletal lesions
Skull vault (frontal, parietal, occipital bones): 12 Skull base, orbit, temporal bone: 7 Spine: 18 Pelvic bone: 12 Tibia: 7 Humerus: 5 Mandible: 3 Rib: 7 Femur: 6 Clavicle: 2 Scapula: 2	LN: 5 Thymus: 5 CNS: 3 Liver: 2 Spleen: 2 Lung: 1	On skeletal survey None On bone scan (n = 4) Pelvic bone, humerus, rib and radius On WB-MRI (n = 2) Humerus, and diffuse bone marrow involvement

A total of 86 skeletal lesions were detected on whole-body imaging modalities in 33 patients (Table 2). The sensitivity for skeletal LCH lesion detection by WB-MRI was significantly higher on that by skeletal survey (98.8% versus 63.0%; p < 0.0001) or bone scan (98.8% versus 46.9%; p < 0.0001), and the sensitivity of skeletal survey was significantly higher than that of bone scan (p = 0.002). There was no significant difference in the mean number of false-positives per patient between the whole-body imaging modalities (skeletal survey versus WB-MRI, p = 0.974; bone scan versus WB-MRI, p = 0.215; skeletal survey versus bone scan, p = 0.971).

**Table 2:** Lesion detectability of skeletal survey, bone scan, and whole-body MRI in patients with Langerhans cell histiocytosis.

	True-positive lesions	False-negative lesions	False-positive lesions	Sensitivity (95% confidence interval)^*^	Mean No. of false-positives per patient^†^
Overall^‡^					
Skeletal survey	56	43	0	56.6% (46.7–66.0%)	0 (0/38)
Bone scan	38	61	4	38.4% (29.4–48.3%)	0.11 (4/38)
WB-MRI	98	1	2	99.0% (93.2–99.9%)	0.05 (2/38)
Skeletal lesions^§^					
Skeletal survey	51	30	0	63.0% (52.0–72.7%)	0 (0/33)
Bone scan	38	43	4	46.9% (36.4–57.8%)	0.12 (4/33)
WB-MRI	80	1	1	98.8% (91.8–99.8%)	0.03 (1/33)

WB-MRI = whole-body magnetic resonance imaging.

*For all comparisons, P < 0.017.

^†^For all comparisons, P > 0.017.

^‡^The number of overall lesions: 99 true lesions and 6 false-positive lesions in 38 patients.

^§^The number of skeletal lesions: 81 true lesions and 5 false-positive lesions in 33 patients.

In terms of whole LCH lesions, including both skeletal and extra-skeletal lesions, sensitivity of WB-MRI was significantly higher than sensitivities of skeletal survey (99.0% versus 56.6%; p < 0.0001) and bone scan (99.0% versus 38.4%; p < 0.0001) (Table 2). There were no significant differences in the mean number of false-positives per patient between the whole-body imaging modalities (skeletal survey versus WB-MRI, p = 0.971; bone scan versus WB-MRI, p = 0.423; skeletal survey versus bone scan, p = 0.969).

Among the 81 true-positive skeletal lesions, 30 lesions (37.0%), 43 lesions (53.1%) and 1 lesion (1.1%) were missed on skeletal survey, bone scan, and WB-MRI respectively (Table S4 in the Supplementary Materials). The most common region of missed lesions was the pelvic bone on both skeletal survey and bone scan. Among the 18 true-positive extra-skeletal lesions, 13 lesions (72.2%) were missed on skeletal survey and all 18 lesions (100%) went undetected on bone scan. All extra-skeletal lesions were detectable on WB-MRI.

### Accuracy of Risk Stratification using the different Whole-body Imaging Modalities

On the basis of the final risk group, 16 patients (34.8%) were classified into SS-LCH without MBL or CRL, 20 patients (43.5%) into SS-LCH with MBL or CRL, 8 patients (17.4%) into MS-LCH without RO, and two patients (4.3%) into MS-LCH with RO.

**Figure 2:**
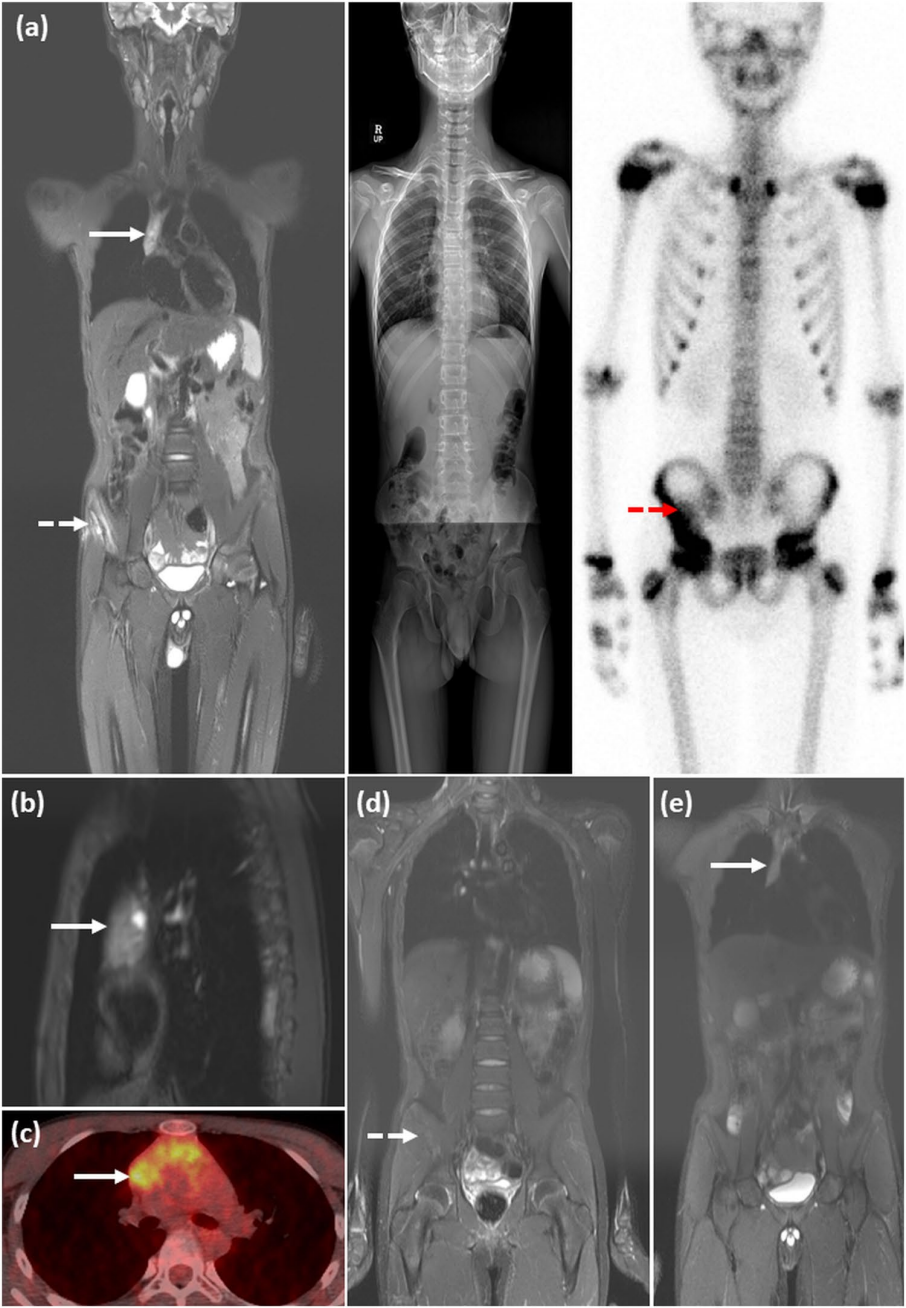
A representative case of an 11-year-old girl with a pathologically-confirmed LCH lesion in the right iliac bone. (**a**) Coronal STIR image from the WB-MRI (left), trunk and lower extremity images of skeletal survey (middle), and anterior view of bone scan (right), obtained for the initial evaluation of the extent of the disease, are shown. A known LCH lesion was detected on WB-MRI and bone scan (dashed arrows), but not on the skeletal survey, probably because of overlying bowel contents. WB-MRI also revealed heterogenous signal intensity with a tiny cystic portion in a slightly enlarged thymus (solid arrow), which was well-visualized on the sagittal STIR image (**b**), suggesting thymic involvement of LCH. However, thymic involvement could not be detected on the skeletal survey or bone scan. (**c**) PET-CT scan showing heterogenous increased FDG uptake in the thymus (arrow), raising the possibility of thymic involvement of LCH. (**d**,**e**) Follow-up WB-MRI after 6 months of treatment showing decreased size of the lesion in the right iliac bone (dashed arrow, **d**), and the near-normalized size and signal intensity of the thymus (arrow, **e**). On the risk stratifications, the patient was under-classified as single system LCH without multiple bone lesions or CNS risk lesions on the skeletal survey and bone scan, but correctly classified as multisystem LCH without risk organ involvement on WB-MRI.

**Figure 3:**
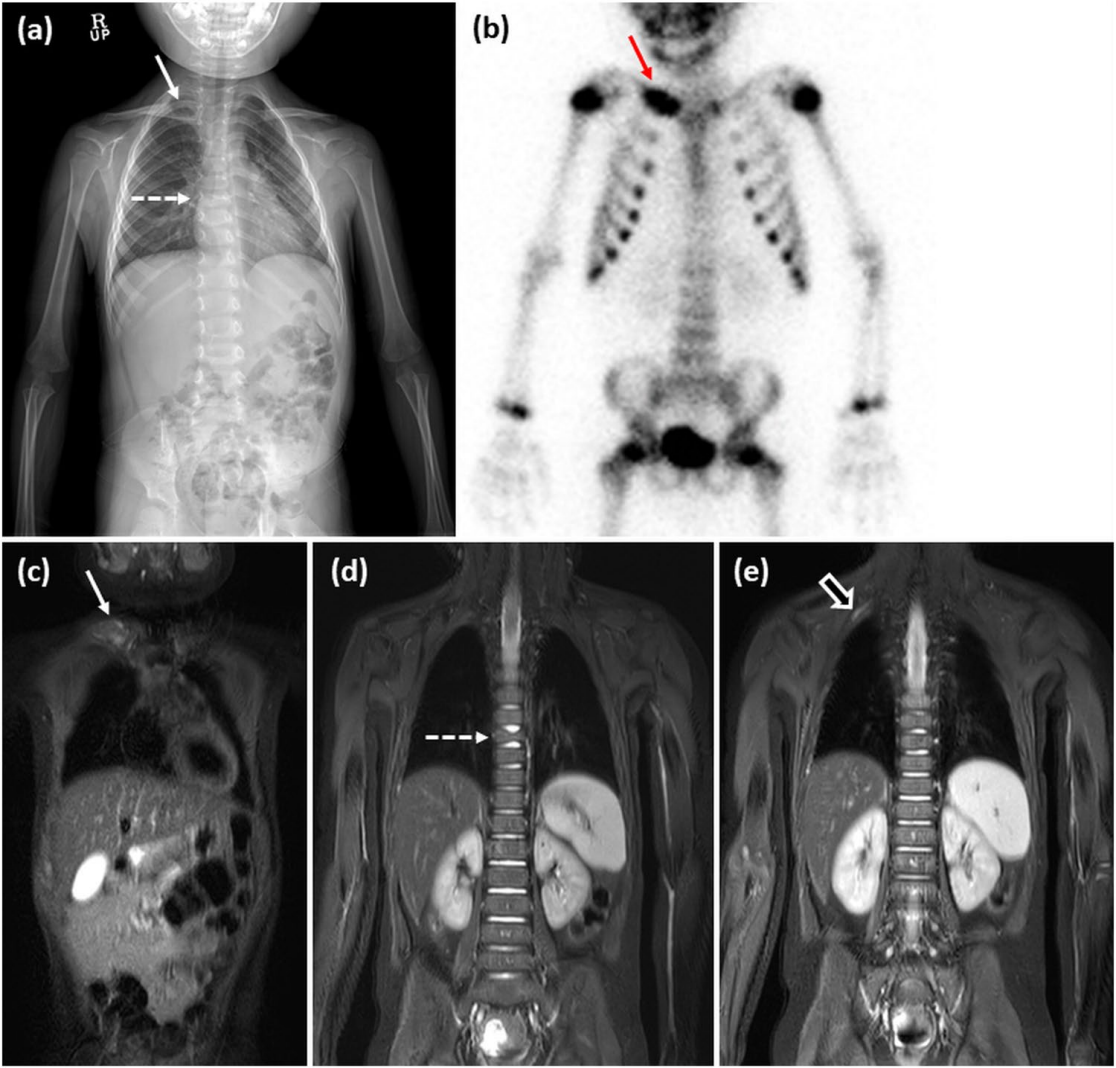
Skeletal survey (**a**), bone scan (**b**) and STIR coronal images of WB-MRI (**c**–**e**) of a 6-year-old boy with a pathologically-confirmed LCH lesion in the right clavicle. The lesion in the clavicle (solid arrow) was detected in all three imaging modalities (**a**–**c**); however, the lesion in the T8 vertebral body, appearing as a partially collapsed vertebral body, was noted only on skeletal survey (**a**) and WB-MRI (**d**), and the lesion in right first rib was noted only on WB-MRI (**e**). Although an additional lesion in the right first rib was detected on WB-MRI but not by the skeletal survey, the risk stratifications by WB-MRI and skeletal survey were identical for single system LCH with multiple bone lesions or CNS risk lesions.

Table 3 shows comparison of risk groups classified according to the reference standard and each whole-body imaging modality. There was no significant difference in the concordance rate of the risk stratification between the three whole-body imaging modalities (concordance rate: 91.3% in skeletal survey, 82.6% in bone scan, and 97.8% in WB-MRI; overall p-value 0.066). Agreements in risk stratification assessed according to the reference standard and each whole-body imaging modality showed almost perfect agreement when using skeletal survey (weighted kappa of 0.88 with 95% CI of 0.75–1.00) and WB-MRI (weighted kappa of 0.98 with 95% CI of 0.93–1.00), and substantial agreement when using bone scan (weighted kappa of 0.66 with 95% CI of 0.44–0.88; Table 3). Representative cases are shown in Figs 2 and 3.

**Table 3:** Comparison of risk groups classified according to the reference standard and each whole-body imaging modality in patients with Langerhans cell histiocytosis.

Risk group (reference standard)	Risk group assigned on each imaging study	Skeletal survey	Bone scan	WB-MRI
SS-LCH without MBL or CRL (n = 16)	SS-LCH without MBL or CRL*	16	15	16
	SS-LCH with MBL or CRL		1	
SS-LCH with MBL or CRL (n = 20)	SS-LCH without MBL or CRL	2	3	
	SS-LCH with MBL or CRL^*^	18	17	20
MS-LCH without RO (n = 8)	SS-LCH without MBL or CRL	1	1	
	SS-LCH with MBL or CRL	1	1	
	MS-LCH without RO^*^	6	6	7
	MS-LCH with RO			1
MS-LCH with RO (n = 2)	SS-LCH without MBL or CRL		2	
	MS-LCH with RO^*^	2		2
Concordance rate (95% CI)^†^		91.3% (79.0–96.7%)	82.6% (68.9–91.1%)	97.8% (86.1–99.7%)
Weighted Kappa (95% CI)		87.5% (75.0–100%)	65.6% (43.7–87.5%)	97.6% (93.0–100%)

^*^Correctly classified patients on whole-body imaging modalities.

^†^Overall P = 0.066.

Note: Dedicated imaging studies of specific regions obtained before the diagnosis of LCH and the sites of pathologically-confirmed LCH lesions are referred to in each session of risk stratification.

SS-LCH = single system LCH; MBL = multiple bone lesions; CRL = central nervous system risk lesions (CRL); MS-LCH = multisystem LCH; RO = risk organ involvement.

## Discussion

This study revealed that in comparison with skeletal survey and bone scan, WB-MRI has a significantly higher sensitivity for the detection of LCH lesions, without a significant increase in the number of false-positives per patient. Although the differences between the imaging modalities were not statistically significant, WB-MRI showed a trend towards higher accuracy in the risk stratification of patients with newly-diagnosed LCH compared with skeletal survey and bone scan. This would ultimately help in the planning of appropriate treatment and determining the prognosis in such patients.

Evaluation of the extent of LCH involvement remains difficult because of the absence of a gold standard diagnostic modality. The guideline by the project Euro-Histo-Net 2008 suggested performing a chest radiograph and skeletal radiograph survey for the evaluation of LCH^24^. It was also mentioned that other imaging techniques such as bone scan, PET scan, or MRI are not an alternative to the standard skeletal survey, because the diagnostic value of these imaging modalities in LCH is still under study. However, in our study, we found that the sensitivity of skeletal survey for overall LCH lesions was unsatisfactory at 56.6%. Even considering that skeletal survey aims to detect skeletal lesions and is limited in the evaluation of extra-skeletal lesions, the sensitivity of skeletal survey for skeletal LCH lesions was disappointing, at 63% in our study. In particular, we found that LCH lesions in pelvic bone and vertebrae, which commonly overlapped with other overlying structures, were often missed on skeletal survey. For extra-skeletal LCH lesions, laboratory findings and physical examination can be used to assess the involvement of the liver or spleen; however, evaluation of thymic involvement is difficult. Radiographs can be of help in detecting the enlargement of the thymus with an irregular contour. In our study, two of five patients with thymus involvement showed thymic involvement on skeletal survey, but it was not found in the remaining three patients. In this regard, skeletal survey, which is recommended by the current guideline for clinical work-up in patients with LCH^24^, might be insufficient to evaluate the exact extent of LCH involvement.

Bone scan, which is often additionally used to evaluate the extent of skeletal lesions in patients with LCH, also showed disappointing results. In our study, bone scan showed a sensitivity of 38.4% for the detection of overall LCH lesions, and a sensitivity of 46.9% for skeletal LCH lesions, which is lower than that of skeletal survey and WB-MRI. Bone scan was insufficient for the detection of all extra-skeletal lesions.

PET-CT is another option for determining the extent of LCH in the initial evaluation and follow-up, because it can assess the physiological activity of the LCH lesion^29^. Previous studies have showed PET-CT to have a greater accuracy in the evaluation of disease extent in patients with LCH compared with skeletal survey and bone scan^3–5,29^. However, the exposure to ionizing radiation limits the use of PET-CT, as patients with LCH require multiple imaging studies to monitor treatment response and detect possible recurrence during the course of their disease. Furthermore, LCH often involves pediatric patients who are more sensitive to the ionizing radiation. PET-CT also has intrinsic limitations in the evaluation of LCH lesions in the CNS and lung^3,29^.

WB-MRI has been applied to a wide range of oncologic indications, including LCH, as it has a great capacity for the characterization of soft tissue lesions with the absence of exposure to ionizing radiation. Only two previous studies have assessed the diagnostic accuracy of WB-MRI in patients with LCH, and these only included a small number of patients; 14 WB-MRIs in 6 patients^9^, and 43 WB-MRIs in 9 patients^8^. These studies reported that WB-MRI showed higher detectability of LCH lesions than skeletal survey and bone scan. Our study also revealed WB-MRI to have excellent lesion detectability, with a sensitivity of 99.0% for overall LCH lesions, and a sensitivity of 98.8% for skeletal LCH lesions. Although MRI may have a tendency to give false-positive results because of its high sensitivity, we found no significant difference in the false-positive rates between WB-MRI, skeletal survey, and bone scan in our study. In addition to higher lesion detectability, WB-MRI does not expose patients to radiation unlike skeletal survey and bone scans. Although LCH can occur at any age, young children are more commonly affected than adults, and multiple imaging acquisitions are required during long follow-up periods. Hence, it is important to choose an imaging modality that does not expose LCH patients to radiation. Nevertheless, WB-MRI has disadvantages such as higher cost and longer acquisition times, which often require anesthesia in young children.

As management and prognosis depend on the risk group to which patients with LCH are classified, accurate pre-treatment risk stratification through whole-body imaging is critical for deciding on the treatment plan and determining the prognosis. Although there have been studies on the diagnostic accuracy of whole-body imaging on initial evaluation and follow-up in LCH patients, no study has evaluated the effect of whole-body imaging on the risk stratification of LCH patients. In this study, we found no significant differences in the accuracy of risk stratification between the three whole-body imaging modalities. However, WB-MRI tended to show a higher concordance rate and agreement with the reference standard than skeletal survey and bone scan. Considering the excellent lesion detectability of WB-MRI, there may be further room to improve the accuracy of WB-MRI in the risk stratification of patients with LCH. Further multi-institutional prospective studies are needed to investigate the clinical significance and cost effectiveness of WB-MRI for risk stratification in patients with LCH.

This study has several limitations. The retrospective design of the study means there may have been several confounding and bias factors. However, LCH is a very rare disease and WB-MRI is not yet routinely applied in clinical practice. Thus, the preliminary results in this study should be confirmed by a future well-designed prospective study. The sample size was small, although it was larger than those of previous studies. Selection bias may be present because only patients who underwent all three whole-body imaging modalities were included in the study. As this study included only patients with LCH, which means there were no true-negative lesions, we were unable to assess specificity. To overcome this shortfall, the mean number of false-positives per patient was evaluated. Since skeletal survey and bone scan have limited roles in the evaluation of extra-skeletal lesions, PET scans would be more relevant than WB-MRI. However, PET scans are not used often in our institution because of the risk posed by radiation exposure, especially for pediatric patients. Thus, a comparison of WB-MRI and PET was not possible in our study. Further studies comparing WB-MRI and PET are needed to justify the use of these imaging modalities and to assess cost effectiveness with LCH patients. Because of the nature of LCH with multisystem involvement, it was not possible or appropriate to biopsy all suspected lesions. Therefore, the final diagnosis of suspected LCH lesions was made via a multidisciplinary approach based on the results of clinical and imaging follow-up by reviewing of electronic medical records and serial follow-up imaging data.

WB-MRI showed higher sensitivity in the detection of LCH lesions than skeletal survey and bone scan, while maintaining a similar number of false-positives per patient. WB-MRI, skeletal survey, and bone scan showed comparable accuracy in the initial risk stratification of patients with LCH, although WM-MRI did show a non-significant trend towards the highest accuracy.

## Electronic supplementary material

 Supplementary Information

## Data Availability

The datasets generated during and/or analysed during the current study are available from the corresponding author on reasonable request.
