# Supplementary material for: Comparison of whole-body MRI, bone scan, and radiographic skeletal survey for lesion detection and risk stratification of Langerhans Cell Histiocytosis
Source: Sci Rep. 2019 Jan 22;9:317. doi: 10.1038/s41598-018-36501-1 (PMC6342958; doi:10.1038/s41598-018-36501-1)
Supplement: Supplementary file 1 — Supplementary Information [file 41598_2018_36501_MOESM1_ESM.docx]

**Comparison of whole-body MRI, bone scan, and radiographic skeletal survey for lesion detection and risk stratification of Langerhans Cell Histiocytosis.**

Jeong Rye Kim. MD^1^, Hee Mang Yoon, MD, PhD*^2^, Ah Young Jung, MD^2^, Young Ah Cho, MD, PhD^2^, Jong Jin Seo, MD, PhD^3^, Jin Seong Lee, MD, PhD^2^.

***Affiliations of the Authors:***

^1^ Department of Radiology, Dankook University Hospital, Cheonan-si, Chungcheongnam-do, South Korea.

^2^ Department of Radiology and Research Institute of Radiology, Asan Medical Center, University of Ulsan College of Medicine, Seoul, South Korea.

^3^ Department of Pediatrics, Asan Medical Center Children’s Hospital, University of Ulsan College of Medicine, Seoul, South Korea.

***Corresponding Author:***

Hee Mang Yoon, MD, PhD.

Department of Radiology and Research Institute of Radiology

Asan Medical Center, University of Ulsan College of Medicine

88 Olympic-ro 43-gil, Songpa-gu, Seoul 05505, Korea

Tel.: 82-2-3010-0906; Fax: 82-2-476-4719

E-mail: [espoirhm@gmail.com](mailto:espoirhm@gmail.com), hmyoon@amc.seoul.kr

**SUPPLEMENTARY MATERIALS**

The criteria used for evaluating the involvement of LCH on imaging findings based on previous studies is as follows: On the skeletal survey, osteolytic or mixed osteolytic and sclerotic lesions, vertebra plana or decreased height of vertebrae, enlarged hepatic or splenic shadow, and enlarged thymic shadow with an irregular contour were considered as LCH lesions. On bone scans, a focal increased radioisotope uptake in the skeleton was considered to be an LCH lesion. On WB-MRI, the following were considered as LCH lesions: (1) focal bone lesions with various degrees of perilesional edema presenting as low signal intensity on T1-weight imaging (WI), high signal intensity on STIR, and enhancement on contrast enhanced images, (2) focal lesions with enhancement in an extra-skeletal organ (although focal lesions showing typical imaging features of other diseases such as hepatic hemangioma were not considered as LCH lesions), (3) lymph node enlargement with a short diameter larger than 1 cm, (4) thymic enlargement with signal change, and (5) hepatomegaly and splenomegaly.

Table S1. Pulse sequence parameters for the whole-body MRI.

| Field strength | 3 Tesla |  |  |  | 1.5 Tesla |  |  |  |
| --- | --- | --- | --- | --- | --- | --- | --- | --- |
| Pulse sequence | STIR COR | STIR SAG | T1 SE COR | Post-contrast T1 mDIXON COR | STIR COR | STIR SAG | T1SE COR | Post-contrast T1 FS COR  eTHRIVE |
| TR (msec) | 5660 | 4245 | 542 | 4 | 3769 | 3297 | 525 | 5.2 |
| TE (msec) | 60 | 60 | 10 | 0 | 71 | 79 | 12 | 2.5 |
| TI (msec) | 220 | 220 | NA | NA | 165 | 165 | NA | NA |
| No. of averages | 1 | 1 | 1 | 1 | 2 | 2 | 2 | 1 |
| Slice thickness (mm) | 5 | 5 | 5 | 1.6 | 5 | 5 | 5 | 3.2 |
| Gap (mm) | 1 | 1 | 1 | 0 | 1 | 1 | 1 | NA* |
| ETL | 50 | 50 | 3 | 2 | 32 | 32 | 5 | 32 |
| Band width  (Hx/pixel) | 375 | 322 | 522 | 1221 | 465 | 369 | 515 | 306 |
| Acquisition Matrix | 344 × 272 | 392 × 209 | 380 × 251 | 296 × 238 | 312 × 212 | 288 × 213 | 308 × 230 | 328 × 327 |
| Flip angle (degree) | 90 | 90 | 90 | 10 | 90 | 90 | 90 | 10 |

STIR: Short tau (inversion time) inversion recovery; COR: coronal; SAG: sagittal; T1: T1-weighted image; SE: spin echo; mDIXON: modified DIXON; FS: fat-suppressed; eTHRIVE: enhanced T1 high resolution isotropic volume excitation (T1-weighted three-dimensional interpolated gradient echo sequence, Philips); TR: repetition time; TE: echo time; TI: inversion time; ETL: echo train length: NA: non-applicable.

* overlapping of 1.6 mm was applied.

Table S2. The extent of LCH involvement in 46 patients.

|  | Single system LCH (n = 36/46, 78.3%) | Multisystem LCH (n = 10/46, 21.7%) |
| --- | --- | --- |
| Age (year) | 9.9 ± 13.1 (0.3–55) | 10.7 ± 13.7 (0.1–46) |
| Sex | Male 19; Female 17 | Male 2; Female 8 |
|  | Skeletal lesions only (n=32)  Extra-skeletal lesion only (n=4)  Skin (n=3)  Lung (n=1) | Extra-skeletal lesion only (n=4)  Skeletal and extra-skeletal involvement (n=6) |

Note. Unless otherwise specified, the data are the number of patients.

Table S3. The distribution of LCH involvement in 46 patients.

| Skeletal lesions  (n = 38/46, 82.6%) | Extra-skeletal lesions (n = 14/46, 30.43%) |
| --- | --- |
| Skull vault (frontal, parietal, occipital bones): 13/46  Skull base, orbit, temporal bone: 8/46  Spine: 13/46  - Extraosseous spinal canal soft tissue formation: 7  Pelvic bone: 8/46  Tibia: 6/46  Humerus: 5/46  Mandible: 3/46  Rib: 4/46  Femur: 4/46  Clavicle: 2/46  Scapula: 2/46 | Skin: 7/46  LN: 5/46  Thymus: 5/46  CNS: 3/46  Liver: 2/46  Spleen: 2/46  Parotid: 1/46  Lung: 1/46 |

Note. Unless otherwise specified, the data are the number of patients.

**Table S4. Distribution of missed lesions on skeletal survey (n = 43), bone scan (n = 61) and WB-MRI (n = 1).**

|  | Missed lesions on skeletal survey (n = 43) | | Missed lesions on bone scan (n = 61) | | Missed lesions on WB-MRI (n = 1) | |
| --- | --- | --- | --- | --- | --- | --- |
| Distribution | Skeletal lesions  (30/81, 37.0%) | Extra-skeletal lesions  (13/18, 72.2%) | Skeletal lesions  (43/81, 53.1%) | Extra-skeletal lesions  (18/18, 100%) | Skeletal lesions  (1/87, 1.1%) | Extra-skeletal lesions  (0/18, 0%) |
| Specific organ/bone | Pelvic bone: 6  Spine: 5  Tibia: 5  Humerus: 3  Mandible: 2  Rib: 3  Femur: 3  Scapula: 1  Skull vault (frontal, parietal, occipital bones): 1  Skull base, orbit, temporal bone: 1 | LN: 5  Thymus: 3  CNS: 3  Liver: 2 | Spine: 8  Pelvic bone: 8  Skull vault (frontal, parietal, occipital bones): 7  Tibia: 5  Humerus: 4  Femur: 4  Skull base, orbit, temporal bone: 3  Mandible: 2  Rib: 2 | LN: 5  Thymus: 5  CNS: 3  Liver: 2  Spleen: 2  Lung: 1 | Femur: 1 | None |
